# Phase versus amplitude sorting of 4D‐CT data

**DOI:** 10.1120/jacmp.v7i1.2198

**Published:** 2006-02-21

**Authors:** Nicole M. Wink, Christoph Panknin, Timothy D. Solberg

**Affiliations:** ^1^ Department of Radiation Oncology David Geffen School of Medicine at University of California Los Angeles, Peter Morton Medical Building, Suite B265 Los Angeles California 90095 U.S.A.; ^2^ Siemens AG Medical Solutions Henkestrasse 127 D‐91052 Erlangen Germany; ^3^ Department of Radiation Oncology University of Nebraska Medical Center 987521 Nebraska Medical Center Omaha Nebraska 68198 U.S.A.

**Keywords:** 4D‐CT, RCCT, gated radiotherapy, respiratory motion, computed tomography

## Abstract

Image quality of CT scans suffers when objects undergo motion. Respiratory motion causes artifacts, which prevents adequate visualization of anatomy. Four‐dimensional CT (4D‐CT) is a method in which image reconstruction of moving objects is retrospectively gated according to the recorded phase information of the monitored motion pattern. Although several groups have investigated the use of 4D‐CT in radiotherapy, little has been detailed with regard to the sorting method. We present a new retrospective gating technique with sorting based on the amplitude of the motion trace. This method is compared to previously developed methods that sort based on phase. A 16‐slice CT scanner (Sensation 16, Siemens Medical Solutions, Erlangen, Germany) was used to acquire images of two phantoms on a motion platform moving in two dimensions. The motion was monitored using a strain gauge inserted inside an adjustable belt. A 180° interpolation was used for reconstruction after gating. Significant improvement using the amplitude‐sorting technique was observed, particularly when testing nonperiodic motion functions.

PACS numbers: 87.59.Fm, 87.53.Kn, 87.57.Ce

## I. INTRODUCTION

Motion due to respiration causes artifacts in CT scanning, which results in a skewed size, shape, and density of objects in the image.^(^ ^1^ ^–^ ^4^ ^)^ In diagnostic imaging, patients are often instructed to hold their breath for the duration of the scan to alleviate these problems. This approach has two shortcomings. First, a long breath‐hold places increased burden on patients, many of whom are unable to do so for an extended length of time.^(5)^ Second, the position of anatomical structures in the lung and diaphragm region changes with respiration, and it is different during a full inspiration breath‐hold than it is during normal respiration. Further, the administration of radiation therapy cannot be performed under breath‐hold due to the duration of the procedure. Therefore, the ability to acquire images that reflect the position of anatomical structures as a function of respiration during free breathing is highly desirable.

In four‐dimensional CT (4D‐CT), the patient is allowed to breathe freely while the respiration trace is recorded for the duration of the CT scan.^(^ ^5^ ^–^ ^10^ ^)^ The time of image acquisition is recorded along with the respiration trace for subsequent correlation of image data with respiratory phase. This correlation allows images that were acquired at the same phase of respiration for all *Z* positions (the superior/inferior patient axis) to be combined into a phase‐specific volume. Doing this for all phases of the respiratory cycle yields complete information about the respiratory motion pattern of the entire volume.

The order and method in which the image data are reconstructed and sorted into proper phase groups is important. Several investigators have proposed a method in which the image data are first reconstructed and subsequently sorted.^(^ ^6^ ^–^ ^10^ ^)^ However, with a finite sampling, this will yield reconstructed slices that were not acquired precisely at the desired phase of respiration and the desired *Z* position, but slightly off in either space or time or both. This necessitates combining adjacent slices that were acquired at slightly different phases of respiration, producing spatial misalignment throughout the volume, and compromising image quality. One group has proposed reconstructing the image data after the sorting process.^(5)^ This method is not restricted to the location of the reconstructed slices and therefore eliminates phase misalignment throughout the image volume.

The majority of the published 4D‐CT techniques have used a time‐based sorting method for retrospectively gating the image data.^(^ ^5^ ^–^ ^10^ ^)^ In this method, the phase position is determined by specifying a percentage of the period of each cycle. As will be demonstrated, data sorted in this manner are subject to misalignment due to varying slopes, periods, and amplitudes in the respiration trace. Rietzel et al.^(9)^ have proposed an amplitude‐sorting method in which a constant amplitude on the respiratory curve is chosen to designate the phase position. Although this method eliminates the possibility of misalignment, it also increases the potential for missing phase sets. If a certain amplitude is not reached in all cycles of the recorded respiration trace, then that phase set cannot be created without a gap in the volume.

We present a new amplitude‐based sorting method in which the reference phase is determined by specifying a percentage of the amplitude of each cycle. This method is compared to the previously published time‐based sorting method. The ability to obtain phase‐specific image volumes for all phases of respiration with minimal misalignment is beneficial for viewing CT images of freely breathing patients with minimal motion artifacts.

## II. METHODS AND MATERIALS

### A. Acquisition

A 16‐slice CT scanner (Siemens Medical Systems, Erlangen, Germany) was used with a helical scanning protocol for 4D‐CT. The scanner is equipped with a direct anode cooling X‐ray tube, enabling scanning for extended periods of time. A new protocol was installed on the scanner to facilitate scanning at a pitch of 0.1 for heavily overlapped data acquisition. Sorting and reconstruction of all 4D‐CT data was performed off‐line using software developed in‐house.

**Figure 1:**
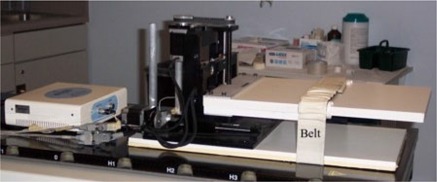
Motion platform used to simulate breathing patterns with the motion‐monitoring system. Adjustable belt is placed around the platform to monitor the AP motion direction.

Two phantoms were placed on a platform (Newmark Systems, Mission Viejo, CA) that moves under computer control using high‐precision stepper motors.^(11)^ In this study, the platform was programmed to move simultaneously in the superior‐inferior (*z*‐axis) and anterior‐posterior (*y*‐axis) patient axes. Motion was monitored using a strain gauge inside an adjustable belt (Anzai Medical, Tokyo, Japan) placed around the motion platform (Fig. 1). A differential RS422 signal was sent from the CT scanner to the Anzai system to indicate X‐ray start and stop times, thus allowing correlation of image acquisition with the respiration trace.

### B. Gating

**Figure 2:**
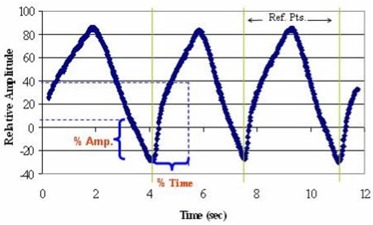
Pictorial description of percentage‐amplitude and percentage‐time gating methods on a nonperiodic, constant amplitude motion pattern. Reference points separate each cycle in the motion function.

A new gating method was implemented for the helical 4D‐CT procedure. The location of the midpoint of the desired phase image data was determined by taking a percentage of the peak‐to‐peak distance in each cycle. Each cycle was separated at the trough position on the respiration curve. In this context, a 50% phase would correspond to the peak amplitude, or end inhale bin. An illustrative example of the phase sorting and amplitude based methods on a nonperiodic motion pattern is shown in Fig. 2.

Retrospective binning was performed at the sinogram level, prior to reconstruction. Ten phases were used throughout all the studies. However, this system is capable of reconstructing any arbitrary number of phases without adding any extra scan time or dose.

The method of time‐based sorting was compared to the percentage‐amplitude gating method by plotting the phase position (midpoint of image data) on every cycle of the recorded motion function for every phase. The consistency in relative amplitude was observed because of its direct correlation with image set alignment.

### C. Phantom Study

**Figure 3:**
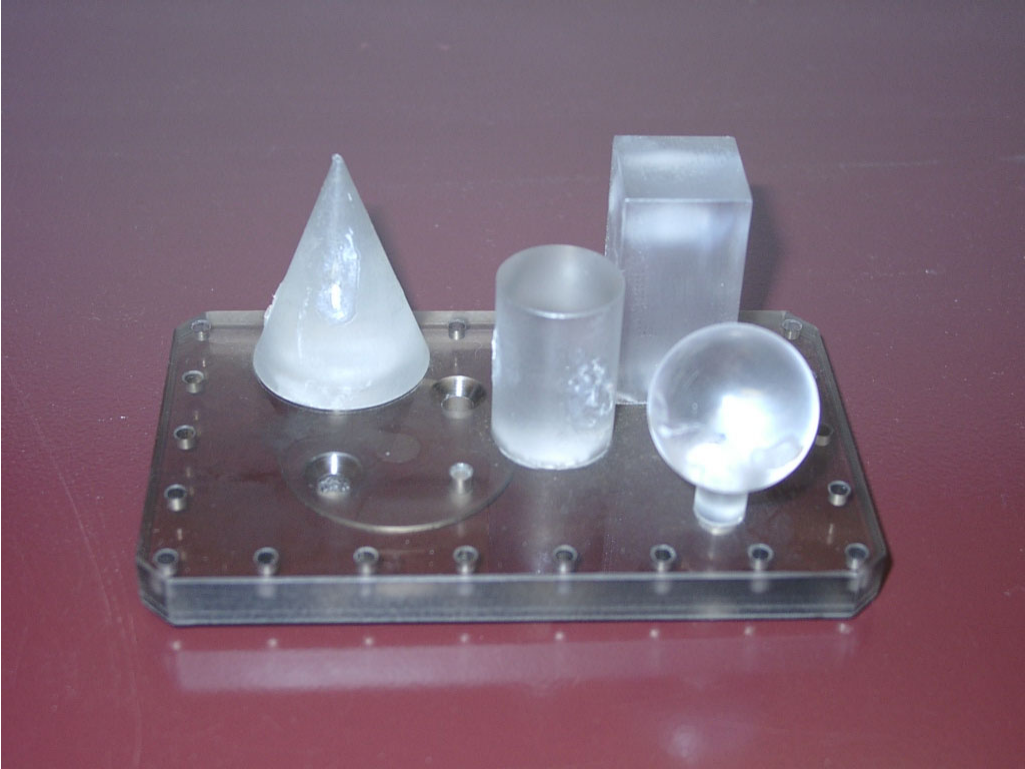
Radiosurgery QA phantom including conical, cylindrical, rectangular, and spherical test objects. This phantom was used for gating method comparisons.

Two different phantoms were imaged for analysis and comparison of each 4D‐CT sorting method. A high‐contrast radiosurgery QA phantom consisting of a sphere, a box, a cylinder, and a cone inside an artificial skull is shown in Fig. 3. The American College of Radiology CT accreditation phantom consisting of two identical but separated spherical beads (both 0.28 mm in diameter) was also used.^(12)^ The adjustable belt on the system monitored primarily the anterior‐posterior (AP) direction of motion.

**Figure 4:**
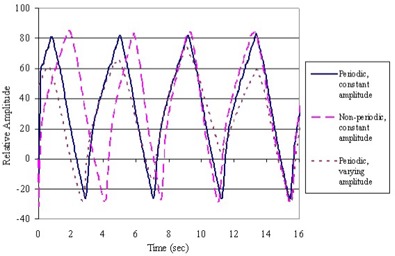
Plot of the three different motion functions programmed for gating method comparison. Periodic and nonperiodic patterns with and without varying amplitudes are diagrammed.

Scans were acquired using a periodic motion function consisting of a peak‐to‐peak superior‐inferior distance of 4 cm, AP distance of 1 cm, and period of 4.2 s to accurately mimic physiologically relevant motion patterns.^(^ ^1^ ^,^ ^3^ ^,^ ^13^ ^–^ ^19^ ^)^ Scans of the stationary phantom were acquired with the same scanning parameters for comparative purposes. In addition, a nonperiodic motion profile was used, with periods ranging from 2 s to 4 s, and peak‐to‐peak amplitudes from 0.5 cm to 4 cm. The three motion profiles are plotted in Fig. 4.

A standard technique of 100 kV and 400 effective milliampere seconds (mAs) was used for each scan. Detector collimations of 0.75 mm and 1.5 mm were used with a 0.5‐s rotation time. In every case, the maximum scan length, limited by a raw data file size of 2 GB, was acquired. For 4D‐CT imaging, scans of moving phantoms were acquired at a pitch of 0.1 and were reconstructed with a 180° interpolation algorithm.^(20)^ For nongated imaging, scans of stationary and moving phantoms were acquired at a pitch of 0.5, the lowest pitch available for nongated scans. The 0.5 pitch scans were reconstructed with a 2.0‐mm slice width and 1.0‐mm increment, while the 0.1 pitch scans used a 1.2‐mm increment and a 2.0‐mm slice width. The scans performed on each phantom are listed in Table 1.

**Table 1:** Scan protocol for all phantom studies

Scan#	Motion	Collimation (mm)	Rotation time (s)	Pitch	Gated
1	static	1.5	0.5	0.5	
2	sinusoidal	1.5	0.5	0.5	
3	sinusoidal	0.75	0.5	0.1	✔
4	sinusoidal	1.5	0.5	0.1	✔
5	irregular 1 and 2	1.5	0.5	0.1	✔

## III. RESULTS

The largest difference between the phase and amplitude‐based gating methods was observed in the constant amplitude, nonperiodic motion patterns. As expected, no significant differences were observed in scans performed using the periodic motion pattern with varying amplitudes. Therefore, the following results comparing the two gating methods are from those scans using the constant amplitude, nonperiodic motion pattern.

**Figure 5:**
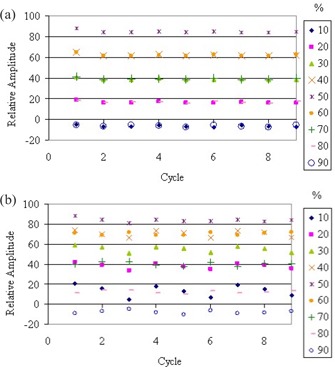
Comparison of (a) percentage‐amplitude and (b) percentage‐time gating methods. Each point represents the midpoint of a block of image data used to construct a phase specific image set. The *y*‐axis is the relative amplitude of the monitored motion pattern, while the *x*‐axis represents the elapsed cycles on the recorded motion trace. The legend distinguishes each phase of motion (i.e., 50% is end inhale). It is important to note the consistency in relative amplitude from cycle to cycle.

The midpoint of the block of image data used to construct each phase‐specific image set is plotted for every cycle of a recorded respiratory curve (Fig. 5). The *y*‐axis represents the relative amplitude of the monitored motion pattern. The legend distinguishes each phase of motion (i.e., 50% is end inhale). The *x*‐axis represents the elapsed cycles on the recorded motion trace. To construct a 4D dataset, the blocks of image data from one phase of motion for every cycle recorded would then be combined. It is important to note that the relative amplitude of each phase from cycle to cycle should be consistent in order to avoid spatial misalignment in the images.

The plots show that the percentage‐amplitude gating method (Fig. 5(a)) results in a more consistent phase location over time, particularly at the phases of maximum motion (midinhale/midexhale). The correlation coefficient $\left(R^{2}\right)$ to a linear fit of the midpoints for each phase is much higher (representing linearity) when reconstructing with the amplitude‐sorting method than with the phase‐sorting method (Table 2. The differences between the gating methods are smallest at peak and trough positions since the least distance is traversed at these points.

**Table 2:** Coefficient of correlation for linear fit on midpoints for each phase and sorting method

Bin	% Amplitude $R^{2}$	% Time $R^{2}$
10	0.14	0.071
20	0.26	0.067
30	0.31	0.12
40	0.31	0.047
50	0.30	0.12
60	0.28	0.050
70	0.24	0.045
80	0.17	0.0039
90	0.051	0.00080
Average	0.23	0.059

**Figure 6:**
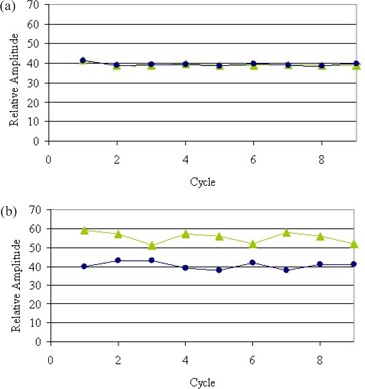
Comparison of (a) percentage amplitude and (b) percentage time gating methods on the 30% and 70% phase sets only, showing the phase location over nine cycles on a nonperiodic, constant amplitude motion pattern. These plots demonstrate the effect of inhale and exhale slope changes on phase location.

It is also apparent that each mirror phase set (i.e., midinhale and midexhale) corresponds more accurately with its counterpart when using the amplitude‐sorting method. An example of the 30% and 70% phase positions for each sorting method is shown in Fig. 6. It is evident that the phase‐sorting method is much more susceptible to inhale and exhale slope differences than the amplitude‐sorting method.

## IV. DISCUSSION

Retrospective gating of image data before reconstruction has many advantages. Unlike other techniques, it is not limited in terms of the location of the reconstructed slices. Rather, each image is reconstructed at the exact desired phase and spatial location. It is not dependent on the reconstruction increment or spacing between slices. If gating is performed after reconstruction, the slices must be close together to reduce the potential for large phase position shifts from cycle to cycle.

The percentage‐amplitude sorting method is a more reliable alternative to other published techniques. The amplitude‐sorting method detailed by Rietzel et al. is optimal for phase location consistency from cycle to cycle, but may not provide an image set for all phases of respiration in a variable‐amplitude breathing pattern. The phase position for each cycle in the percentage‐time sorting method is affected significantly by amplitude, period, and inhale and exhale slopes, which makes this technique susceptible to misalignment in image location throughout a phase‐specific image set. Although the percentage‐amplitude method presented here is also susceptible to irregularities in amplitude from cycle to cycle, it adequately handles changes in period and slope. It is important to note that any misalignments in image location would directly affect the quality of phase‐specific image sets.

The programmed motion functions were chosen to most accurately mimic physiologically relevant motion patterns based on various published abdominal motion studies.^(^ ^3^ ^,^ ^14^ ^–^ ^19^ ^,^ ^21^ ^,^ ^22^ ^)^ While a wider range of motion speed could be easily be accommodated by the platform, a 4.2‐s period was implemented. If the detector rows move past the image plane before one full respiratory cycle plus one fan angle is complete, a gap in the image dataset occurs. This situation, encountered when scanning in helical mode, enforces a period limit on the monitored motion function.^(5)^

The 4D‐CT approach used in this paper is based on external monitoring; however, internal motion may not correlate directly with the external monitor.^(23)^ The incorporation of internal target motion into future respiratory motion correction techniques would reveal the degree to which external monitoring can accurately reflect internal motion. Monitoring internal motion in patients would require fluoroscopy or surgically implanted markers; however, it would provide precise answers for 4D verification.

## V. CONCLUSIONS

The use of 4D information has numerous applications in furthering radiotherapy treatment planning. Based on the experiments shown, it potentially reduces motion blurring in CT images of therapy patients that are unable to hold their breath. In addition, monitoring the location of the target provides the extent of movement in any direction and the trajectory it follows. This information can be used for target volume definition to ensure complete coverage of the target. Knowing the trajectory can also be helpful when tumor tracking or gated therapy is desired.

Several advances have been made to ameliorate the process of acquiring a 4D image dataset. The results presented here demonstrate a novel retrospective sorting method based on the full respiration curve that provides an image set at every phase. The benefits of this method over established phase sorting techniques have been demonstrated on multiple phantoms.

## ACKNOWLEDGMENTS

This work was supported by grant #03‐028‐01‐CCE from the American Cancer Society and Siemens.
